# A complementary marriage of perspectives: understanding organizational social context using mixed methods

**DOI:** 10.1186/s13012-014-0175-z

**Published:** 2014-11-23

**Authors:** Rinad S Beidas, Courtney L Benjamin Wolk, Lucia M Walsh, Arthur C Evans, Matthew O Hurford, Frances K Barg

**Affiliations:** Center for Mental Health Policy and Services Research, Perelman School of Medicine, University of Pennsylvania, 3535 Market Street, Floor 3, Philadelphia, PA 19104 USA; Department of Behavioral Health and Intellectual disAbility Services, 1101 Market Street, Philadelphia, PA 19104 USA; Community Behavioral Health, 801 Market Street, Philadelphia, PA 19107 USA; Department of Family Medicine and Community Health, Perelman School of Medicine, University of Pennsylvania, 3620 Hamilton Walk, Philadelphia, PA 19104 USA

**Keywords:** Mixed-methods, Organizational social context, Organizational social context measure, Inductive, Deductive

## Abstract

**Background:**

Organizational factors impact the delivery of mental health services in community settings. Mixed-methods analytic approaches have been recommended, though little research within implementation science has explicitly compared inductive and deductive perspectives to understand their relative value in understanding the same constructs. The purpose of our study is to use two different paradigmatic approaches to deepen our understanding of organizational social context. We accomplish this by using a mixed-methods approach in an investigation of organizational social context in community mental health clinics.

**Methods:**

Nineteen agencies, representing 23 sites, participated. Enrolled participants included 130 therapists, 36 supervisors, and 22 executive administrators. Quantitative data was obtained via the Organizational Social Context (OSC) measure. Qualitative data, comprised of direct observation with spot sampling generated from agency visits, was coded using content analysis and grounded theory. The present study examined elements of organizational social context that would have been missed if only quantitative data had been obtained and utilized mixed methods to investigate if stratifying observations based on quantitative ratings from the OSC resulted in the emergence of differential themes.

**Results:**

Four of the six OSC constructs were commonly observed in field observations (i.e., proficiency, rigidity, functionality, stress), while the remaining two constructs were not frequently observed (i.e., resistance, engagement). Constructs emerged related to organizational social context that may have been missed if only quantitative measurement was employed, including those around the physical environment, commentary about evidence-based practice initiatives, leadership, cultural diversity, distrust, and affect. Stratifying agencies by “best,” “average,” and “worst” organizational social context impacted interpretation for three constructs (affect, stress, and leadership).

**Conclusions:**

Results support the additive value of integrating inductive and deductive perspectives in implementation science research. This synthesis of approaches facilitated a more comprehensive understanding and interpretation of the findings than would have been possible if either methodology had been employed in isolation.

## Introduction

A burgeoning body of research has emerged to suggest that organizational factors are particularly important characteristics that impact delivery of mental health services for youth in the community [1–3]. The organizational literature suggests the importance of an individual’s social context on one’s attitudes, beliefs, and subsequent behavior around adoption of innovation [3,4]. In the case of youth mental health services, the most important social context refers to the organizations within which treatment is delivered (i.e., organizational social context) [5]. Two important constructs, culture and climate, contribute to organizational social context. Organizational culture refers to shared employee perceptions around “the behavioral expectations and norms that characterize the way work is done in an organization” ([6], p. 858), whereas organizational climate refers to shared employee perceptions around “the psychological impact of their work environment on their own personal well-being” ([5], p. 64). Organizational culture and climate have been associated with provider turnover [3,7], quality of services [8], sustainment of adoption of new practices [7], and youth mental health outcomes [8,9]. The gold standard assessment of organizational culture and climate is the Organizational Social Context (OSC), a quantitative measure [10] developed over the past 35 years [11]. Items are based on qualitative work, expert review, and empirical testing (Glisson, personal communication, 2014).

Measurement continues to present a thorny challenge in implementation science [12,13]. As efforts to translate research to practice have become a national priority, more diverse methodologies such as mixed-methods approaches are becoming more sophisticated and increasingly utilized [14,15]. Mixed methods allow for quantitative data (e.g., surveys) to be integrated with qualitative data (e.g., interviews) to allow for a more comprehensive understanding of organizations (e.g., [15]). When research is done from a purely etic perspective, the outsider or “expert” guides the research. In contrast, research that aims to understand the insider’s view takes an emic perspective [16]. Mixed-methods research can ideally result in a synergistic effect in which the combination of emic and etic perspectives is greater than either individual contribution [17].

The purpose of our study is to demonstrate the use of mixed methods to deepen our understanding of organizational social context. We accomplish this by juxtaposing the use of a validated and reliable measure with an inductive real-world set of observations to examine organizational social context in community mental health clinics. We take three different and complementary approaches to understand organizational social context within community mental health clinics. We began with a deductive approach using the OSC. As a standardized, validated instrument, the OSC is based on the premise that the constructs of proficiency, rigidity, resistance, engagement, functionality, and stress are central to characterizing and quantifying organizational culture and climate, two major components of organizational social context. The instrument’s purpose is to identify the presence and strengths of the key constructs. However, the instrument is limited to measuring only the constructs identified by experts as important to organizational social context. Second, we conducted direct observations with spot sampling of 23 agencies which refers to observation and recording of behavior with periodic randomly selected visits to the context of interest [18]. We used content analysis to identify whether we could observe real-world examples of the six OSC constructs within our field notes. Finally, we used a grounded theory approach to determine if there were additional factors in our field notes, not covered by the OSC constructs, which might contribute toward organizational social context. Agencies were stratified based on their organizational social context scores (“best,” “average,” and “worst”) to determine if different constructs were present based on the quality of organizational social context.

## Methods

### Participants

We used purposive sampling to recruit the 29 largest child-serving agencies in Philadelphia, which together serve approximately 80% of youth receiving publically funded mental health care. Of these 29 agencies, 18 (62%) agreed to participate. Additionally, one agency involved in evidence-based practice (EBP) efforts asked if they could participate, resulting in a final sample of 19 agencies. Several agencies had multiple locations, resulting in 23 sites, 130 therapists, 36 supervisors, and 22 administrators. Each site (*N* = 23), rather than each agency (*N* = 19), was treated as a distinct organization because of different leadership structures, locations, and staff. Going forward, we will refer to the site as “agency”.

### Procedure

All procedures were approved by associated Institutional Review Boards. Qualitative data were gathered through direct observation with spot sampling and were recorded as field notes during agency visits. These observations were collected as part of a study to measure use of EBP in community mental health agencies (see [19]). As part of this project, the research team visited 23 agencies for approximately 2 h to administer a battery of self-report measures and to collect observational data. Following the visit, both the first and third author recorded field notes based on observations of the therapists and supervisors with whom they administered measures. The majority of the field notes documented direct observation of the group. However, a small minority of interactions were one-on-one through short informal conversations that occurred while visiting with the group. All field notes included comments on the physical atmosphere (e.g., temperature, building appearance), the professional atmosphere (e.g., collegiality among staff), and general impressions about the visit as these were *a priori* constructs of interest.

The OSC quantitative measure was collected as part of the 2-h visit mentioned above along with a number of other measures. Therapists completed the OSC in their referent group as indicated by the measure developers. Supervisors and executive administrators completed the measure separately. Participants received $50.00 for participating in the larger study.

### Measures

#### Demographics

We asked all participants to provide information on their background (e.g., age, gender, ethnicity).

#### Organizational social context

The OSC measurement system [10] is a 105-item measure of the social context of mental health and social service organizations. The OSC measures organizational culture and organizational climate with a mean of 50 and a standard deviation of 10. The OSC measurement model defines organizational culture as comprised of three dimensions—proficiency, rigidity, and resistance—whereas organizational climate is also comprised of three dimensions—engagement, functionality, and stress [10].

The OSC has norms based on a normative sample of 100 mental health service organizations nationally and has strong psychometric properties [20]. This measure was completed by therapists, supervisors, and executive administrators. In this sample, the measure demonstrated adequate internal consistency across subscales (Cronbach’s alpha = .71–.95) [21].

### Data analytic plan

#### Quantitative analysis

To calculate each dimension of the OSC, clinician responses by agency were aggregated, after ensuring that aggregation was indicated through the use of the *rwg* statistic [22,23]. Intra-group agreement was excellent (mean *rwg* = .95), supporting aggregation of clinician, supervisor, and administrator response. We elected to include supervisors and administrators in our sample because it minimally impacted the data and allowed us to retain all of the agencies in our OSC sample.

Following procedures outlined by Glisson and colleagues, we used latent profile analysis (LPA) to generate a continuous score demarcating summative agency organizational social context ranging from 1 to 3 [24]. This composite OSC score is derived from the six profiles for each agency based on the probability weighted sum of class membership in one of three empirically derived OSC profiles (“worst,” “average,” and “best”). These three profiles were originally identified by using LPA on OSC scores from a national sample of 100 children’s mental health agencies [10]. Using LPA, we applied estimates from the national sample to the agencies in this study to calculate the probability that each of the agencies was a member of the three empirically derived classes. Agencies were assigned composite OSC scores by multiplying the possibility that the agency was a member of each class by the value for that respective class and summing the products resulting in a variable that ranged from 1.00 (most negative profile; “worst”) to 3.00 (most positive profile; “best”) [24], allowing us to classify the 23 agencies into the following types of organizational social context: “best” (*N* = 7), “average” (*N* = 5), and “worst” (*N* = 11).

#### Qualitative analysis

Our qualitative analysis proceeded in two phases. The first (RSB) and second (CBW) authors independently conducted a content analysis of the field notes. We examined field notes from each agency to identify the presence or absence of examples of constructs from the OSC (i.e., proficiency, resistance, rigidity, engagement, functionality, stress). Next, we used a grounded theory analysis of the field notes to identify additional features that might affect organization social context within the agency. We (RSB, CBW) conducted an independent line-by-line reading of each field note and created a set of codes as they emerged from the texts. We used the inter-rater reliability function in QSR Nvivo 10.0 (Kappa = .94) to ascertain agreement.

Constructs that emerged from our grounded theory process had some overlap with OSC constructs. For example, phrases in the field notes that referred to both leadership and the interactions between leaders and staff could be double coded under both “leadership” and “rigidity.” However, we felt that our direct observations suggested the importance of creating a distinct construct that documented the impact of leaders on organizational social context that was not fully captured under the rigidity construct. See Table 1 for the codebook.

**Table 1:** **Code book**

**Construct**	**Definition**	**Examples**
Proficiency^a^	“Proficient cultures are characterized by expectations that service providers will place the wellbeing of each client first and by expectations that individual service providers will be competent and have up-to-date knowledge. Representative items include ‘Members of my organizational unit are expected to be responsive to the needs of each client’ and ‘Members of my organizational unit are expected to have up-to-date knowledge.’ The Proficiency scale consists of both competency and responsiveness items. Competency describes the emphasis that is placed on training, up-to-date knowledge, and expectations of excellence in skills and abilities. Responsiveness describes the extent to which service providers are expected to meet the unique needs of individual clients.”	“One of the therapists was talking about participating in a motivational interviewing training today.”
		“The practice principles of the Department of Behavioral Health and Intellectual disAbility Services were in the waiting room as well. We were in a conference room upstairs. There was a large rectangular table that we sat around. It was quiet. Core values of the agency were listed on the walls (effective communication, commitment, quality of services, trust, respect, professionalism (knowledge), and empowerment). There was also a determination poster and an accreditation (expired) certificate on the wall.”
		“Certain therapists seemed to have a difficult time understanding what they were being asked to do.”
		“Therapist said that she felt like therapists were just hired to fill empty spots, not based on merit or on their attitude towards evidence-based practices. She said in her interview for this job, they basically just asked if she had worked with kids and didn’t ask much about what kinds of therapy they do.”
Rigidity^a^	“Rigid cultures are characterized by service providers having less discretion and flexibility in their work; limited input into key management decisions; and being controlled by many bureaucratic rules, and regulations. Representative items include ‘I have to ask a supervisor or coordinator before I do almost anything’ and ‘The same steps must be followed in processing every piece of work.’ The rigidity scale includes both centralization and formalization. Centralization indicates the degree to which power and decision making are in the hands of relatively few while formalization characterizes the level of procedural specifications that guide work-related interactions among members of an organizational unit. Organizations that are highly centralized and highly formalized emphasize control with little individual autonomy or participative decision making.”	“Both therapists checked everything off on the training survey. At first it seemed like they didn’t understand, but then the second therapist reported worries that it would get back to the higher ups that she hadn’t heard of certain practices. We assured her that we would be careful about what information we reported.”
		“One therapist got up and left after he realized that it was a voluntary study.”
		“Another therapist said he ‘wanted to keep his job’ and wanted to know if I would be sharing results with administration. I said no but that he didn’t have to fill anything out that he didn’t want to.”
		“One male therapist sat with his arms crossed at the table. When I asked him if he wanted to participate, he said that he did not, and I told him he could leave.”
Resistance^a^	“Resistant cultures are characterized by expectations that service providers will show little interest in change or in new ways of providing service, and that service providers will suppress any opportunity for change. Representative items include, ‘Members of my organizational unit are expected to not make waves’ and ‘Members of my organizational unit are expected to be critical.’ Resistance includes items to assess apathetic and suppressive behavioral expectations. Apathetic items assess the level of resignation and inactivity towards change while suppression items mark expectations of criticism and opposition that undermine openness and innovation.”	“[Participant] thinks people implementing evidence-based practices are not giving other treatments a chance (mainly psychodynamic)…Said the reason that we haven’t found that psychodynamic treatment is effective is a) we haven’t done enough research, b) it can’t be measured in the same way as cognitive-behavioral therapy.”
		“Likes evidence-based practices a lot. Is getting training on his/her own in several. He/she thinks that some of the older therapists don’t like evidence-based practices because the treatments are new and seem complicated and are too structured.”
Engagement^a^	“Engaged agencies are characterized by employee perceptions that they are able to personally accomplish many worthwhile things in their work, remain personally involved in their work, and be concerned about their clients. Representative items include, ‘I feel I treat some of the clients I serve as impersonal objects (reverse coded)’ and ‘I have accomplished many worthwhile things in this job.’ These items include both personalization and personal accomplishment items. Personalization items indicate the degree to which organizational members feel connected and involved with their clients. Personal accomplishment assesses perceptions of efficacy in dealing with clients and positive emotions related to success with clients.”	“Therapist said that she had a case load of ‘75-85’ clients. Said that she was really stressed out, that she ‘loved her kids’ but felt overwhelmed.”
		“[Participant] said his supervision isn’t from psychologists or psychiatrists (they have them in the building) and doesn’t focus on how to best treat children and families. Said the supervision is focused on them making more money, getting in their paperwork, and seeing clients. Said that he has told them that he can’t see any more, but they don’t care. Said that when he shares with his supervisor that a client has shown improvement, she doesn’t really care if it means that he didn’t make the client quota for the week.”
Functionality^a^	“Functional climates are characterized by employee perceptions that they receive the cooperation and help from coworkers and administration required to do their job, have a clear understanding of how they fit in, and can work successfully within their organizational unit. Representative items include, ‘This agency provides numerous opportunities to advance if you work for it,’ ‘My job responsibilities are clearly defined,’ and ‘There is a feeling of cooperation among my coworkers.’ The functional scale includes items for growth and advancement, role clarity, and workgroup cooperation. These include perceptions that continual development and advancement will occur, that expectations for one’s work behavior are clearly presented, and that organizational unit members will assist and aid in one’s work when needed.”	“Everyone was very friendly, from the receptionist to the therapists. Everyone interacted nicely with one another and there seemed to be an atmosphere of collegial respect. Therapists were all very chatty and jovial.”
		“Overall, the atmosphere was quite collegial and people seemed to enjoy being in the same room together. There was a lot of laughter and engagement among the staff.”
		“Therapists were mostly on time and respectful of one another. They joked about their days and seemed to interact nicely as colleagues. Talked about their kids openly with us and joked about their clients. Everyone from the security guards to our contact person were very welcoming.”
Stress^a^	“Stressful climates are characterized by employee perceptions that they are emotionally exhausted from their work, pulled in different directions, and unable to get the necessary things done. Representative items include, ‘I feel like I am at the end of my rope,’ ‘Interests of the clients are often replaced by bureaucratic concerns (e.g., paperwork),’ and ‘The amount of work I have to do keeps me from doing a good job.’ Stress is identified by emotional exhaustion, role conflict, and role overload items. Respectively, these include perceptions of feeling overwhelmed, of experiencing multiple conflicting demands, and having impossible amounts of work to accomplish.”	“Also she told us that there used to be six therapists here when she started (five years ago), but now there are only three.”
		“People asked a few questions but were generally quiet during the data collection process. The intercom was bothering a few of the therapists who felt frustrated that it kept going off and interrupting them. One therapist said she wanted to go home so that she didn’t have to deal with it anymore.”
		“Subway shook the building and made a lot of sound periodically.”
Leadership^b^	Leadership refers to interactions with leaders at the agencies such as supervisors, clinical directors, and executive directors. Typically, this will refer to references around therapist behavior when leaders leave the room or references to how frequently therapists interact with leadership. Note, simply mentioning the leader is not enough (i.e., the clinical director was welcoming); it should refer to an interaction between the researcher and the leaders or the leaders and staff. This interaction could be hypothetical (i.e., I never meet one on one with my executive director) or actual (i.e., leader encourages staff to be honest on the questionnaires).	“When I brought up that they would be rating their executive director, many of them said they had only met [her] once or had never interacted with her.”
		“Once the clinical director left the room, the therapists were much rowdier and were calling out numbers on the Organizational Social Context measure saying that they were funny.”
		“The therapists were playful with one another and their supervisor. They were less talkative when the other administrators joined us.”
		“Many of them noted that they had no relationship with their executive director and didn’t even know what her name was. One person said, ‘I wouldn’t know who she was if she walked in this room.’”
Physical space^b^	Physical space refers to any mention of the building, room, or part of the city that the agency is in.	“The agency was well lit and sunny and the waiting room was inviting. The receptionists were friendly. The training room had a TV, computer, and projector set up, and we were all seated at a big boardroom table with chairs. Walls look freshly painted. Conference room had long square table with nice leather chairs. Had abstract paintings.”
		“Outside of building was brick and looked a bit run down. Inside was clean and looked like parts of the building had been updated somewhat recently. We held the meeting on the 2nd floor main conference room. It was a clean, nicely lit room with large windows on one side. There was a long, rectangular table with approximately enough seating for 20. There was also a kitchen to the side and a projector system.”
Culture/diversity^b^	Culture/diversity refers to mention of cultural impacts on the agency visit. This does not refer to organizational culture, rather it refers to situations such as agencies that primarily serve a particular cultural group (e.g., Latino/a) or where language barriers were evident.	“Staff seemed to be segregated by language in terms of cliques.”
		“There was also a language barrier. Most of the therapists in this agency were [*non-English speaking*], and had difficulty understanding the questionnaires.”
Affect^b^	Affect refers to observations around participant affect during the agency visit. For example, often therapists laughed during completion of the OSC or seemed anxious around completion of leadership ratings. Any emotion words (“laughter, hesitant, anxious”) would qualify.	“Lots of laughter around Organizational Social Context question about fatigue. Said they were always tired.”
		“Supervisor pulled me aside and was almost tearful and asked if she could mail in her forms and that she was feeling overwhelmed after completing the measures because she has been in meetings all day. She also told me the Organizational Social Context measure made her feel depressed and burnt out.”
		“Therapists were quiet in the beginning but became much rowdier as the clinical director left the room. One therapist was especially talkative and stated, ‘The Organizational Social Context measure makes me think I should reconsider the field I am in.’”
Distrust^b^	Distrust refers to observations or statements about participant distrust. This distrust could be targeted at the researcher, Department of Behavioral Health and Intellectual disAbility Services, or the leadership at their agency.	“Also they asked me several times if I really wanted them to be honest.”
		“Lots of hesitation around agreeing to consent for the study. One therapist in general had a lot of questions regarding confidentiality and risk management (e.g., suicidality of participants). Seemed like none of them wanted to sign the consents until everyone else did.”
		“Another therapist said he ‘wanted to keep his job’ and wanted to know if I would be sharing results with administration. I said no but that he didn’t have to fill anything out that he didn’t want to.”
Initiatives^b^	Any mention of city initiatives (i.e., Beck Initiative, Prolonged Exposure, Trauma Initiative, Evidence-Based Practice and Innovation Center; EPIC) would qualify.	“No one had heard of EPIC.”
		“They also noted they did the Beck [Cognitive Therapy] training two years ago.”
		“Everyone had heard of EPIC and was excited for us to be visiting.”
		“One of the supervisors hadn’t heard of any initiatives.”

^a^The definitions of the first six dimensions replicate the definitions of the dimensions from the Organizational Social Context measure (OSC) [10]. ^b^The remaining dimensions in the table are not assessed with the OSC and emerged using grounded theory.

#### Mixed-methods analysis

The structure of our mixed-methods analysis was QUAN + QUAL (i.e., simultaneous collection and analysis of both data types, giving both equal weights). The function of our analysis was convergence (i.e., do all data collection strategies answer similar questions?) and complementarity (i.e., elaboration of the quantitative data using qualitative data) [25]. The OSC LPA quantitative score was used to stratify the qualitative data so that comparisons could be made across the three types of agencies in each construct.

## Results

### Sample demographics

On average, agencies employed 11.65 (SD = 9.80) therapists. Table 2 provides demographic information about therapist and supervisor participants. Administrators (*N* = 22) were split equally between male (50%) and female (50%). Of the total group of administrators, 15% identified as Hispanic/Latino. Subsequently, when asked to identify ethnicity/race, executive administrators self-identified as Asian (9.1%), African American/Black (18.2%), White/Caucasian (54.5%), multiracial (9.1%), or missing ethnicity/race (9.1%). Highest educational degree attained included bachelor’s degree (9.1%), master’s degree (50.0%), doctoral degree (31.8%), and missing (9.1%).

**Table 2:** **Therapist and supervisor demographics—descriptive statistics**

**Variable**	**Therapist frequency (%) or mean (standard deviation)**	**Supervisor frequency (%)or mean (standard deviation)**
Gender^a^		
Male	23%	22.2%
Female	76%	69.4%
Transgender	1%	0%
Hispanic/Latino^a^		
Yes	20%	19.4%
No	75%	69.4%
Ethnicity^a^		
Asian	4.9%	0%
Black or African American	22%	16.7%
White	54.5%	55.6%
Hispanic/Latino	10.6%	13.9%
Multiracial	4.1%	0%
Other	4.1%	2.8%
Academic Background^a^		
Bachelor’s degree	3.8%	0%
Master’s degree	82.3%	75.0%
Doctoral degree	9.2%	13.9%
Licensure status^a^		
Yes	24.6%	52.8%
No	39.2%	25%
In process	31.5%	13.9%
Primary theoretical orientation^a^		
Psychodynamic	7.7%	5.6%
Behavioral	4.6%	5.6%
Cognitive	3.8%	5.6%
Cognitive-behavioral	38.5%	36.1%
Systemic	15.4%	8.3%
Object relations	2.3%	2.8%
Other	3.1%	0%
Eclectic	20.0%	27.8%
Age	38.09 (11.63)	46.09 (10.44)
Years of clinical experience	6.89 (6.84)	8.71 (5.68)
Years at current agency	3.35 (4.65)	7.48 (6.88)
Current caseload	28.79 (22.05)	-
Level of professional burnout	4.23 (2.58)	3.24 (2.35)
Hours of supervision received/provided each week	1.32 (1.21)	8.95 (7.95)

^a^Does not add up to 100% because of missing responses.

### Quantitative OSC results

See Table 3 for quantitative scores presenting the six dimensions of culture and climate as measured by the OSC as well as the categorization for each agency (i.e., best, average, worst) as calculated by the LPA.

**Table 3:** **Organizational Social Context scores by agency**

**Site**	***N***	**Ratio 1**	**Ratio 2**	**Prof.**	**Rigidity**	**Resistance**	**Engagement**	**Function.**	**Stress**	**LPA**
A	8	.56	.38	52	53	56	57	56	51	Avg
B	8	.75	.25	58	56	56	59	75	55	Best
C	14	.27	.13	46	59	65	49	55	58	Worst
D	8	.71	.38	59	64	57	48	65	53	Avg
E	5	.33	.33	21	65	88	37	19	81	Worst
F	10	.53	.20	59	53	61	61	68	57	Best
G	5	.33	.60	59	44	53	62	62	42	Best
H	5	.15	.40	56	69	59	58	70	55	Avg
I	9	1	.22	53	62	63	64	62	55	Worst
J	15	.48	.25	69	54	75	53	85	46	Best
K	5	.6	.40	54	58	68	58	52	68	Worst
L	9	.73	.11	31	49	53	43	58	54	Avg
M	7	1	.14	41	63	80	43	49	73	Worst
N	8	.71	.44	54	67	73	53	62	59	Worst
O	4	.33	.50	65	66	72	54	71	62	Worst
P	4	.5	.50	56	50	64	57	58	61	Avg
Q	7	1	.14	59	60	64	64	68	48	Best
R	7	.44	.43	58	58	65	48	75	59	Worst
S	4	.57	.20	52	79	78	57	56	63	Worst
T	4	.28	.50	40	60	70	44	42	73	Worst
U	15	.65	.27	64	51	53	57	72	52	Best
V	7	.71	.29	56	53	57	60	70	39	Best
W	8	.33	.33	37	64	78	54	50	61	Worst
Mean	7.65	.56	.32	52.09	58.97	65.48	53.89	60.92	57.51	-

Ratio 1 = number of participants in our study/total number of therapists in child outpatient program, Ratio 2 = number of administrators who completed the Organizational Social Context (OSC) measure/total number of participants who completed the OSC; *prof*. proficiency, *funct.* functionality, *LPA* latent profile analysis.

### Qualitative results—content analysis

Content analysis of the field notes revealed “real-world” examples of the OSC constructs (i.e., proficiency, rigidity, resistance, engagement, functionality, and stress).

#### Proficiency

Proficiency was observed through physical representations as well as discussions had with participants at each agency. Physical representations of proficiency were observed through evidence of participation in evidence-based or evidence-informed efforts (e.g., sanctuary principles were hanging on the wall in several agencies), or more generally, posters that were therapeutically relevant (e.g., emotions), or suggested child-friendly environments (e.g., toys).

In a number of agencies, observations of participants elucidated the type of knowledge individuals had. At one agency, a participant had not heard of the government organization responsible for overseeing city mental health efforts. At another agency, a number of therapists noted that they were receiving training in motivational interviewing the next day.

#### Rigidity

Rigidity was primarily observed around leadership-related interactions. First, at a number of agencies, rigidity was reflected in therapist understanding of their participation in this study. Many initially thought that participation was mandatory as it was presented by their leadership, suggesting little discretion and autonomy among therapists. Second, most observations of rigidity revolved around leader behaviors. At one agency, leadership elected to stay in the room despite our request that they complete their surveys in a separate room. Third, a number of therapists voiced concerns about how the information collected would be shared with leadership.

#### Resistance

Relatively few instances of resistance were observed in the field notes. Of those observations, they primarily related to one-on-one conversations with therapists who relayed their concerns which were almost always specific to cognitive-behavioral therapy (CBT), and a sense that those practicing psychodynamic therapies or those with more years of clinical experience were resistant to CBT.

#### Engagement

Few instances of engagement were observed in the field notes from agency visits. Of the two observations, one therapist noted caring very much for her youth clients, but feeling overwhelmed because her case load was very high. Another therapist reflected that his supervisor did not emphasize how to best treat children and families, focusing on money.

#### Functionality

Functionality was observed through the collegial, professional, and respectful relationship among participants observed at the majority of the agencies. The collegial environment observed across most agencies contrasted with a minority of settings where the chaotic environment appeared to undermine functionality or when staff members appeared not to know one another.

#### Stress

Evidence of stress was observed across a number of domains during agency visits, including stress around job security, fatigue, emotional exhaustion, and the physical environment. At a number of agencies, therapists voiced concerns about job security. Typically, these concerns were around recent downsizing. Understandably, participants had some concerns about sharing negative perceptions of their organization given recent cutbacks. Further, fatigue often came up during agency visits, and comments were made about the OSC question related to fatigue. We observed both emotional and physical environment stress. Emotional stress refers to comments and affect around fatigue, feeling overwhelmed, and feeling burned out. Physical environment stress refers to stressors in the physical space such as a building close to a train (i.e., frequent vibrations and noise), cramped quarters, and/or unpleasant working conditions.

### Qualitative results—grounded theory

A grounded theory analysis of the field notes revealed six additional distinct factors beyond those included in the OSC that seemed important to organizational social context. These factors included the physical environment, leadership, participation in initiatives, cultural diversity, distrust, and affect.

#### Physical environment

The majority of observations referred to the neighborhood/location of the agency and condition and cleanliness of the facility. Additional observations described security, technology, and sensory factors that may contribute to the work environment. Note that this construct frequently overlapped with “stress,” but also captured positive aspects of the physical environment, as well as the actual physical layout of the agency.

Neighborhood/location observations spanned desirable and undesirable qualities and included comments about the safety of the neighborhood and the area of town, the type of building (e.g., a converted row home), and comments on convenience (e.g., has its own parking lot) or potential pitfalls of the location (e.g., under the train tracks). With regard to condition and cleanliness, some agencies were perceived as poorly maintained and lacking adequate resources (e.g., meeting rooms were too small to accommodate everyone), while others had dedicated conference space with adequate seating, appeared well kept, and were perceived as clean and welcoming (e.g., child-friendly decorations, values of the agency displayed on posters). At several agencies, a security guard was present at the entrance. Additional physical environment observations included comments about technology (e.g., the conference room was set up with technology) and sensory observations. Temperature and noise (e.g., the intercom repeatedly went off, a train passed and shook the building periodically) were frequently noted.

#### Initiatives

Observations relating to city-sponsored EBP initiatives included participant mention of specific EBP initiatives or training opportunities, experience implementing EBP, and comments about the Evidence-Based Practice and Innovation Center (EPIC), a new center sponsored by the Department of Behavioral Health and Intellectual disAbility Services (DBHIDS) to support EBP in the City of Philadelphia.

At several agencies, therapists and/or leadership mentioned participation in city-sponsored training initiatives (often CBT) or other evidence of participation in initiatives was evident (e.g., trauma informed care binders were visible). Some participants described positive experiences implementing EBPs following participation in an initiative, including feeling like experts following training and consultation in CBT, while others conveyed feeling that particular initiatives were disorganized or required rigid adherence. While most agencies were familiar with EPIC, several exceptions were noted including agencies that had never heard of EPIC and/or were confused or distrustful about what EPIC would accomplish.

#### Leadership

Leadership observations that were not captured in the “rigidity” construct included differences in therapist behavior when supervisors were not present and frequency of contact between therapists and leaders.

Therapist/leader behavior and affect among one another was observed in a number of instances. Most commonly, this was seen as changes in therapist behavior after leaders exited the room (e.g., increased rowdiness) or when therapists were asked to rate certain leaders (e.g., making faces, laughing). At a number of agencies, therapists did not know who the leadership of their agency was. Finally, contact between leadership and therapists was typically captured when therapists mentioned infrequent contact with supervisors or leaders (e.g., only seeing their supervisor two to four times per month).

#### Cultural diversity

Observations relating to cultural diversity were identified at a few agencies that identified as primarily non-English speaking. Most cultural diversity observations were related to spoken and written language, including that therapists were speaking to one another in another language, or that some therapists required translation of paperwork. Occasionally, the research team had difficulty understanding conversation among staff because they communicated with one another in another language.

#### Distrust

These observations primarily consisted of distrust of the research team, agency leadership, and general groupthink. Distrust of research included instances in which participants expressed concern about loss of confidentiality and about the purpose of the research. Distrust of agency leadership captured instances in which participants expressed fear of repercussions if negative perceptions were made known to administration. Finally, several instances of groupthink were coded in which participants waited until others had consented to participate before doing so for themselves, or where no one asked any questions.

#### Affect

Affect was commonly observed around completion of the OSC (i.e., laughter and joking) and when asked to rate supervisors/leadership. Two types of affect were commonly observed: anxiety and laughter. Note that this construct sometimes overlapped with “stress”; however, affect was not fully captured by stress.

With regard to affect, in several instances, individuals were tearful or overwhelmed when considering OSC questions about fatigue and burnout. Faces were made by several therapists when told they would have to rate their agency leadership. Further, many groups of therapists became more lively and rowdy while completing the OSC, calling out questions they found amusing, particularly when leadership was no longer in the room.

### Stratification by “best,” “average,” and “worst” organizational social context

For our mixed-methods analyses, we stratified agencies into “best,” “average,” and “worst” organizational social context based upon by their quantitative score on the LPA. Then, we explored and compared the content generated in each node by agency type (i.e., “best,” “average,” “worst”) to see if differential qualitative content was yielded. Table 4 provides information on how many of the agencies within each type of agency had content coded at that node (i.e., for the “affect” construct, eight out of the eight “worst” agencies had content coded at that node). Table 5 provides examples of qualitative content stratified by organizational social context.

**Table 4:** **Qualitative constructs stratified by “best,” “average,” and “worst” Organizational Social Context agencies**

**Theme**	**Worst (** ***N*** **agencies)**	**Average (** ***N*** **agencies)**	**Best (** ***N*** **agencies)**	**Total number of agencies where construct was observed**
Affect	8	3	5	16
Culture/diversity	1	0	1	2
Distrust	5	2	0	7
Engagement	2	0	0	2
Functionality	11	5	7	23
Initiatives	6	4	4	14
Leadership	10	5	7	22
Physical space	11	5	7	23
Proficiency	9	4	3	16
Resistance	3	0	1	4
Rigidity	6	3	4	13
Stress	9	3	3	15

We used latent profile analysis (LPA) which uses all six dimensions of culture and climate simultaneously to categorize the organizations into “best”, “average,” and “worst” organizational social contexts. This table provides information on our mixed-methods analysis. Each row represents a construct from our qualitative analysis and provides information on how many of each type of agency (“best”, “average,” and “worst” organizational social context) demonstrated evidence of each construct. For example, the construct “affect,” was observed in five of the agencies with “best” organizational social context, three of the agencies with “average” organizational social context, and eight of the agencies with “worst” organizational social context. In the final column, the total number of agencies that a construct was observed within is detailed, so affect was observed in 16/23 of the agencies.

**Table 5:** **Sample field note content stratified by “best,” “average,” and “worst” Organizational Social Context agencies**

**Construct**	**Worst agencies**	**Average agencies**	**Best agencies**
Affect	Supervisor pulled me aside and was almost tearful and asked if she could mail in her forms and that she was feeling overwhelmed after completing the Organizational Social Context measure because she has been in meetings all day.	Faces were made by several therapists when [researcher] mentioned that they would have to rate their leadership.	One therapist was nervous and asked if we were doing the experiment todayand if she needed to prepare.
	The therapists were in general very lively. There was laughter around the question, “I think evidence-based practice is a waste of time and money for this team.”	When the supervisor was asked to leave the room, she asked the therapists to “Be kind.” Lots of laughter around that.	
Leadership	Two of the therapists said that they didn’t know the clinical director very well.	When I brought up that they would be rating their executive director, many of them said they had only met their executive director once or had never interacted with her.	One therapist raised his hand and said that they only had good things to say about their clinical director.
	The therapists were playful with one another and their supervisor. They were less talkative when the other administrators joined us.	Faces were made by several therapists when [researcher] mentioned that they would have to rate their clinical director and executive director.	Cramped, therapists had one common work room, very few therapy rooms.
Stress	[Participant] says old timers are resistant to evidence-based practices because they don’t understand it and are stressed about their jobs.	Therapist offices were small and darkly lit without a lot of posters, decorations, or toys in the rooms.	The intercom was bothering a few of the therapists who felt frustrated that it kept going off and interrupting them.
	Lots of laughter around Organizational Social Context question about fatigue. Said they were always tired.	When the clinical director came to drop off her packet, told us “Sorry if I seem distracted. There is some emergency going on.”	Could hear people walking around the house (as it was an old row home) and secretary kept calling on the intercom for therapists.
	Therapist said that she had a case load of “75-85” clients. Said that she was really stressed out, that she “loved her kids” but felt overwhelmed.		

We used latent profile analysis (LPA) which uses all six dimensions of culture and climate simultaneously to categorize the organizations into “best,” “average,” and “worst” social contexts. This table provides information on our mixed-methods analysis. Each row represents a construct from our qualitative analysis and details the actual observations made about that theme, stratified by type of organizational social context.

Four of the factors (i.e., culture/diversity, distrust, engagement, resistance) were only observed in a few agencies, so we did not explore differential content because there were too few observations to make meaningful interpretations. Five of the factors (i.e., rigidity, functionality, proficiency, initiatives, and physical space) did not result in differential content when stratified by organizational social context. Three of the factors (affect, leadership, stress) demonstrated differential content when stratified by organizational social context (see Table 5 for example content).

#### Affect

Agencies that were rated as “worst” organizational social context appeared to display more instances of affect when compared to “best” or “average” agencies. Specifically, therapists in these agencies demonstrated more negative affect by being more likely to sigh or engage in ironic laughter when completing measures, or grimace when asked to rate their supervisor. However, interestingly, in these agencies, therapists and supervisors were more likely to joke with one another than in “best” or “average” agencies.

#### Leadership

In agencies that were rated as “worst” or “average” organizational social context, we observed marked differences in therapist interactions with the research team when supervisors were not in the room. Further, at these agencies, it seemed like therapists were less likely to have a relationship with upper-level leadership, while more likely to have a close relationship with their direct supervisor. Agencies that were rated as “best” organizational social context seemed to demonstrate evidence of more respectful and bidirectional relationships between therapists and leadership.

#### Stress

Agencies that were rated as “worst” or “average” organizational social context demonstrated more human/emotional stress and related affect when compared to agencies that were rated as “best” organizational social context. For example, typical observations in “worst” and “average” agencies included participants stating that they felt “overwhelmed,” “burned out,” and “fatigued.” Both therapists and supervisors in these agencies appeared to be preoccupied and distracted. Furthermore, in several agencies, the therapists mentioned recent downsizing. Additionally, the physical space was more likely to be described as “uncomfortable” in “worst” and “average” agencies. In “best” agencies, typical observations related to stress and chaos in the physical environment included disruptions such as intercom buzzing.

### Case examples of best, average, and worst organizational social context

To illustrate our findings, we provide case examples from three agencies representing either “best,” “average,” or “worst” organizational social context. These examples are condensed from the full field notes. See Table 3 for quantitative information relating to the OSC.

#### Best organizational social context

Agency J employed 25 therapists providing services to youth; 12 of these therapists were engaged in the study (48%). Therapists predominantly were doctoral level (50%); other educational backgrounds included bachelor’s level (8%) and master’s level (17%). Educational level was missing for 25% of participants.

Agency J was located in a converted row home in a predominantly residential area. The research team met with staff in a room with unfinished floors and mismatched chairs; therapists had to complete paperwork on their laps. Stickers were arranged haphazardly on the walls. Children could be heard in the waiting room crying at times. Overall, the agency was described as “chaotic” but “respectful.” Many therapists were late, would frequently leave the room, and were “constantly talking to each other.” There were primarily [*non-English*] speaking therapists, and therapists were observed interacting primarily with those speaking their primary language. The research team reported there was frequent laughter but that they often did not know what the staff was laughing about because those staff members were speaking [*another language*]. Many therapists required translation of questionnaires. The executive director was described as “very helpful”.

#### Average organizational social context

Agency P had a total of 4 therapists providing services to youth; 2 of these therapists were engaged in the study (50%). One therapist had a bachelor’s degree, the other a master’s degree.

Agency P was located in an “old” building “in a not so nice part of town” with “an old elevator that looked unsafe.” A nearby train could frequently be heard passing by. The building included a range of behavioral health services. The meeting room had group rules on the wall and posters about “feelings.” There was a table with chairs around it and “several of the chairs were broken.” Outside the room, children could be heard crying and playing. The group was described by the observers as “very friendly,” and it was noted that “everyone seemed very collegial and respectful.” The clinical director “seemed really nice and well-respected” and was heard reassuring therapists that they could answer questions freely without concern of repercussions.

#### Worst organizational social context

Agency E had a total of 12 therapists providing services to youth; 4 of these therapists were engaged in the study (33%). Therapists all had a master’s degree.

Agency E was in an “old stone” building that was part of a larger campus with a security guard and several security checkpoints to reach the meeting room. The room contained a table, computer, and projector. The therapists were “quiet and respectful” and “didn’t interact very much with their supervisor.” The therapists “had no questions” about the research. The clinical director asked a lot of questions, and there “seemed to be a lot of distrust around completion of the study.” One therapist arrived late and a member of the research team met with her individually. During this time, the staff member made unsolicited comments about the negative perceptions she has of the agency’s leadership.

## Discussion

The results from this study suggest the additive value of approaching implementation science-related questions, particularly those relating to organizational social context, using mixed methods. Calls have been made to include mixed methods in implementation science [25–27], but empirical demonstrations are less common. We identified constructs within our field notes consistent with our quantitative measurement model using content analysis and also used grounded theory to identify newly emergent constructs which would have been missed without approaching our research question in this manner. This allowed us to demonstrate the convergent and divergent findings that emerged through the use of mixed methods in a study of organizational social context in community mental health clinics.

Given that organizational culture and climate were measured reliably and validly using the OSC, a primary research question had to do with whether we would be able to identify the six elements measured by the OSC in field notes. Four of the constructs were frequently observed (i.e., proficiency, rigidity, functionality, stress). Two of the constructs were not readily observed in agency visits (i.e., resistance, engagement). It is likely that these constructs would be better captured in the use of semi-structured interviews with stakeholders, rather than participant observation, given that when we did observe these constructs, it was within the context of one-one-one conversations with therapists. Interviews can be more effective ways to gather difficult, less visible information [28].

A number of constructs may have been missed if only quantitative measurement was employed such as observations about the physical environment, participation in initiatives, leadership, cultural diversity, distrust, and affect. Three of these constructs, (i.e., physical environment, initiatives, and leadership) can be conceptualized as consistent with the deductive perspective but not wholly captured under deductive constructs. Physical environment was observed at every agency and is a construct that direct observation is well suited to capture. Although this theme had overlap with the stress construct, a number of important additional observations were made around safety, comfort, cleanliness, the sensory experience, and favorable physical environments. Similarly, the initiatives construct had overlap with proficiency, but captured specific comments and opinions around various initiatives sponsored by the City of Philadelphia. Leadership also overlapped with the rigidity construct. An important distinction not captured by rigidity had to do with frontline therapist interactions with leadership. Surprisingly, a number of frontline providers expressed that they had never met agency leadership. We suggest that perhaps these important observations, particularly around leadership and physical space, be integrated into quantitative measurement models of organizational social context. For example, questions related to leadership could include “How often do you interact with your leadership?” and “Do you know your leader’s name?”, whereas questions related to physical space could include “How clean is your physical environment?” and “How comfortable is your work environment?” Further, much discourse around the importance of leadership [29–32] in implementation of innovation suggests the importance of consideration of leadership as a distinct aspect of organizational social context.

The other three constructs related to organizational social context, (i.e., cultural diversity, distrust, and affect) would have been missed completely without using an inductive framework, and suggest the strength and added value of approaching measurement from a mixed-methods perspective [25,26]. Cultural diversity was observed in agencies where primarily ethnic minority youth and families were served; in certain instances, therapists were primarily non-English speaking and had difficulty understanding some of the self-report measures. Themes around distrust emerged at approximately one-third of the agencies visited. Therapists and supervisors made comments indicating distrust around the research team and leadership, suggesting important insights around the researcher and community partner relationship. Finally, affect (e.g., laughter, sadness) was observed in a number of agencies, particularly around completion of the organizational measures. Given these findings, we suggest that these are theoretically important dimensions that should be taken into account alongside organizational social context in future implementation research. We recommend that researchers include these constructs in field notes when collecting quantitative data on organizational social context in the future.

Stratifying agencies by “best,” “average,” and “worst” organizational social context resulted in differential content for the constructs of affect, leadership, and stress. “Worst” agencies demonstrated more displays of affect, both negative and positive from participants when compared to “average” and “best” agencies. It may be that at agencies with poor organizational social context, there may be fewer professional boundaries with regard to affect expression so that therapists are more used to expressing their emotions freely. “Worst” and “average” agencies demonstrated more evidence of poor interactions with leadership, pointing to the importance of organizational interventions that address participant affect and leadership [29]. Finally, “worst” and “average” agencies demonstrated more instances of human stress rather than environmental stress when compared to “best” agencies, as expected [7].

### Limitations

First, the majority of observations obtained herein involved groups versus individual staff members. It is possible that another method, such as individual interviews, would have better facilitated the disclosure of sensitive information (e.g., leadership) [28]. However, group formats are excellent for observing interactions among individuals within a group [14,15]. We were interested in organizational-level constructs rather than individual-level constructs; thus, we believe that observations in the group setting are more relevant. It is possible that individuals who work in positive organizational social contexts may be more likely to feel comfortable expressing concerns about their organization in the presence of coworkers, while those who work in negative organizational social contexts may be more reluctant, making this a potential limitation. However, our experiences suggest that therapists felt comfortable expressing their feelings about their agency, even when they had negative organizational social context ratings. Second, we only spent 2 h at each agency which is a short period of time to fully appreciate the context in which the phenomenon of interest takes place and must be considered a limitation of our study. Moreover, the sample of time in which the observations took place may not be representative of the context at another day or time of day. The interpersonal dynamics between the observer/interviewer and interview can also affect the impressions that are gathered, and intrapersonal factors (e.g., having a bad day) may affect the observations and impressions that are gathered. However, the systematic nature of our observations and analysis (i.e., focus on actors, activities, setting; chronologic nature of observations; field notes that go from wider context to more focused context; immediate processing of field notes, systematic coding and inter-rater reliability checks) helps to address some of these necessary shortcomings. Third, because we did not have 100% of therapists at each agency participating, there is a potential threat to representativeness. Fourth, because administrators were included in the ratings of organizational social context, it is possible that the ratings may have been different if only therapists were included, although we explored the data with only therapists and it was not different.

### Implications

Results of the present study suggest there may be organizational social context challenges to overcome in the City of Philadelphia. At present, there are no system-wide interventions targeting organizational social context across a large service system. Evidence-based interventions to improve organizational social context within agencies exist such as the Availability, Responsiveness, and Continuity (ARC) organizational intervention [33]. An evidence-based intervention strategy, such as the ARC, has the potential to be extended and applied more broadly to an entire system. By targeting systems, rather than individual agencies, a broader impact may be realized.

It is also important to consider how organizational social context is assessed within a system. Typically, organizational social context is measured using quantitative methods. Our results suggest that a combination of participant observation and interviews may facilitate capturing a range of important constructs. A multi-method, multi-informant approach to assessing organizational social context may be recommended, as it has been in other areas of health research. For example, in the assessment of child psychopathology, best practice recommendations include that the perspectives of parents, teachers, and the children themselves be obtained and that, when possible, varying assessment modalities (e.g., self-report and interview) are employed [34,35]. Implementation science research may benefit from a similar model. Using mixed methods may require more resources than utilizing either approach in isolation. While it is possible that this may limit the feasibility of using both approaches in future research, we found that we were able to integrate these perspectives relatively easily and cost-effectively by recording field notes obtained during the quantitative data collection phase of a project.

A number of frontline providers expressed that they did not know individuals in key leadership roles within their agency. The disconnect between providers and leadership is also likely problematic, as leaders have been shown to play an important role in change within an organization [36–39]. Transformational leadership has been associated with innovation climate during implementation, and innovation climate is related to provider attitudes toward EBPs [31]. Situations in which frontline providers and leaders have little interaction may require interventions that target the agency’s culture first (e.g., increasing interactions among members of the organization) before targeting leadership styles and implementation climate. One potential avenue for exploration includes whether agencies that employ fee-for-service therapists have less therapist-leadership interaction.

The constructs of cultural diversity, distrust, and affect were novel and have implications for future research in this area. Given that cultural diversity emerged as a construct, this suggests the need to attend to cultural and language issues in implementation research. For example, it will be important to understand whether current measures of organizational social context are valid for use with therapists for whom English is not the primary language or in agencies implementing EBPs with diverse populations. Distrust around the research team and leadership was observed in a number of agencies. This suggests opportunities may exist to bolster a more collaborative and bidirectional partnership between the community agencies/providers and the researchers involved with EBP implementation, as has been recommended by Chambers and Azrin [40]. Finally, affect (e.g., laughter, sadness) was often observed, particularly around completion of the organizational measures. As the assessment of organizational social context appears to have elicited observable affect in this sample, this may present opportunities for future research to consider the development of an intervention for this purpose. For example, an intervention for clinicians who express feeling overwhelmed or depressed in the context of their organization could include strategies for managing stress and identifying supports in their workplace.

## Conclusion

The present study explored the complementary contributions of inductive and deductive perspectives. Results support the additive value of mixed-method perspectives in implementation science research. Indeed, this synthesis of approaches allowed us to better understand and interpret the findings than would have been possible if the quantitative and qualitative findings were viewed in isolation. Future mixed-methods research in implementation science research is recommended.

## Abbreviations

EBP(s): Evidence-based practice(s)
OSC: Organizational Social Context
DBHIDS: Department of Behavioral Health and Intellectual disAbility Services
CBT: Cognitive-behavioral therapy
EPIC: Evidence-Based Practice and Innovation Center
ARC: Availability Responsiveness and Continuity organizational intervention
